# Balance recovery for lower limb exoskeleton in standing posture based on orbit energy analysis

**DOI:** 10.3389/fbioe.2024.1389243

**Published:** 2024-04-29

**Authors:** Mengze Li, Bi Zhang, Ligang Liu, Xiaowei Tan, Ning Li, Xingang Zhao

**Affiliations:** ^1^ State Key Laboratory of Robotics, Shenyang Institute of Automation, Chinese Academy of Science, Shenyang, China; ^2^ Institutes for Robotics and Intelligent Manufacturing, Chinese Academy of Sciences, Shenyang, China; ^3^ Research Center for Frontier Fundamental Studies, Zhejiang Lab, Hangzhou, China; ^4^ BYD Auto Industry Company Limited, Shenzhen, China

**Keywords:** wearable exoskeleton, balance assistance, rehabilitation, bio-inspired control, model predictive control

## Abstract

**Introduction:** The need for effective balance control in lower limb rehabilitation exoskeletons is critical for ensuring stability and safety during rehabilitation training. Current research into specialized balance recovery strategies is limited, highlighting a gap in biomechanics-inspired control methods.

**Methods:** We introduce a new metric called “Orbit Energy” (OE), which assesses the balance state of the human-exoskeleton system based on the dynamics of the overall center of mass. Our control framework utilizes OE to choose appropriate balance recovery strategies, including torque controls at the ankle and hip joints.

**Results:** The efficacy of our control algorithm was confirmed through Matlab Simulink simulations, which analyzed the recovery of balance under various disturbance forces and conditions. Further validation came from physical experiments with human subjects wearing the exoskeleton, where a significant reduction in muscle activation was observed during balance maintenance under external disturbances.

**Discussion:** Our findings underscore the potential of biomechanics-inspired metrics like OE in enhancing exoskeleton functionality for rehabilitation purposes. The introduction of such metrics could lead to more targeted and effective balance recovery strategies, ultimately improving the safety and stability of exoskeleton use in rehabilitation settings.

## 1 Introduction

Rehabilitation training is crucial for enhancing the quality of life of patients, particularly in restoring capabilities such as standing and walking (Siviy et al., 2023). Standing balance is a core component of rehabilitation training, essential for maintaining independence and safety in daily life activities (Afschrift et al., 2022). Exoskeleton robots, as innovative assistive technologies, have demonstrated unique value in rehabilitation training. Studies have shown that exoskeletons significantly improve the efficacy of standing and walking training, especially for patients with lower limb disabilities, by providing controlled and repetitive training environments (Rodríguez-Fernández et al., 2021; Zhou et al., 2021). Additionally, the use of exoskeletons is associated with multiple health benefits, including improved blood circulation, reflex activities, and bowel and bladder functions (Zhou et al., 2021; Beck et al., 2023). In neurological patients, rehabilitation using exoskeletons has been shown to aid significantly in advancing standing and walking skills while reducing the risk of falls (Lippi and Mergner, 2020; Postol et al., 2021).

To date, there has been notable progress in the development of control technologies for exoskeleton robots (Su et al., 2023), focusing primarily on compliance and interaction. For instance, Zhou et al. emphasized research progress in Lower Limb Rehabilitation Exoskeleton Robots (LLRER), particularly in mechanical design and control technologies (Zhou et al., 2021). Another study specifically analyzed the application of human gait analysis in the design and control of LLRERs (Shi et al., 2019). Additionally, several studies focused on the latest advancements in exoskeleton technology, especially in lower limb motion assistance (Siviy et al., 2023).

The importance of balance control in exoskeletons has been recognized in some research, but a comprehensive exploration of this vital aspect remains lacking (Baud et al., 2021). For example, a study on spinal cord injury patients explored the application of ground-walking exoskeletons in rehabilitation, mentioning gait speed and pain management, but discussions on balance were not comprehensive (Postol et al., 2021). Another research focusing on LLRERs for stroke patients highlighted the importance of providing support and balance in rehabilitation training tasks, yet specific balance control algorithms were not deeply studied (Farkhatdinov et al., 2019). Furthermore, studies on neurological patients using exoskeletons indicated that despite progress in rehabilitating standing and walking skills, challenges such as postural instability and risk of falls highlight the importance of balance control (Lippi and Mergner, 2020).

Only a limited number of studies have focused on enhancing the balance performance of exoskeletons by adjusting joint stiffness or implementing impedance control strategies. For example, Ugurlu et al. proposed (Ugurlu et al., 2015) ankle joint variable stiffness control, Karavas et al. (2013) and hou et al. proposed (Huo et al., 2021) impedance control, Rajasekaran et al. proposed (Rajasekaran et al., 2015) adaptive balance recovery strategy, focuses on specific joints or aspects of balance, and Sugiura et al. proposed (Sugiura et al., 2023a; Sugiura et al., 2023b) support polygon control that assists balance during actions such as standing, kneeling, and sit-to-stand transfers by expanding the support polygon in accordance with shifts in the center of gravity position. Comprehensive approaches that integrate the influence of the wearer and the entire human-exoskeleton system’s dynamics are still evolving (Stegall et al., 2013).

Collectively, these studies suggest that while significant advancements have been made in the application of exoskeleton technology in the field of rehabilitation, especially in enhancing interaction and adapting to human motion, Specialized algorithms designed specifically for standing balance control are relatively scarce. This indicates that ensuring the stability and safety of standing balance remains a key issue to be addressed in the research and application of exoskeleton robots.

Given their structural and functional similarities to bipedal robots, lower limb exoskeletons can benefit from bipedal robots’ balance strategies (Peng et al., 2017). In the field of bipedal robots, notable advancements in stability assessment and balance recovery control have been made (Stephens, 2007; Millard et al., 2009; Stephens and Atkeson, 2010; van der Kooij et al., 2016). For example, Stephens and Atkeson (2010) proposed a Push Recovery Model Predictive Control (PR-MPC) for adjusting stride against external forces, while Wieber (2006) developed a linear MPC scheme for dynamic disturbance compensation. Nishiwaki and Kagami (2009) achieved stable walking with an updateable predictive controller, and Englsberger et al. (2015) introduced subject-time control for three-dimensional DCM trajectories.

The integration of balance strategies from bipedal robots into exoskeleton systems encounters distinct challenges, especially due to the disparities between bipedal robotics and the exoskeleton-human systems. Bipedal robots typically rely on model-based control approaches, necessitating the identification of a model that aptly simulates both bipedal robotics and the exoskeleton-human systems, thereby becoming a pivotal element for the adaptation of successful algorithms. The Flywheel Inverted Pendulum (FIP) model offers a pivotal solution in bridging these disparities. Employed as a prevalent model for balance control, it utilizes the inertia of the trunk and the torques of the lower limbs to negate external perturbations, a strategy that closely mirrors the balance tactics of the human. This approach not only encapsulates the dynamic balance mechanisms inherent to humans but also provides a foundational basis for advancing balance control technologies within exoskeleton robotics.

In this paper, we present a control framework for standing balance in lower limb exoskeleton robots, with ‘Orbit Energy’ (OE) serving as a balance evaluation metric. The framework integrates torque controllers for the ankle and hip joints. The control system, based on the OE and Flywheel Inverted Pendulum model, assesses the human-machine system’s balance state and selects appropriate control strategies. The effectiveness of the algorithm is verified through Matlab Simulink and tests with a physical exoskeleton robot. By examining changes in muscle activity during push-recovery experiments with subjects, we analyze how the proposed method aids in balance recovery.

## 2 Model and orbit energy

### 2.1 Virtual model

**FIGURE 1:**
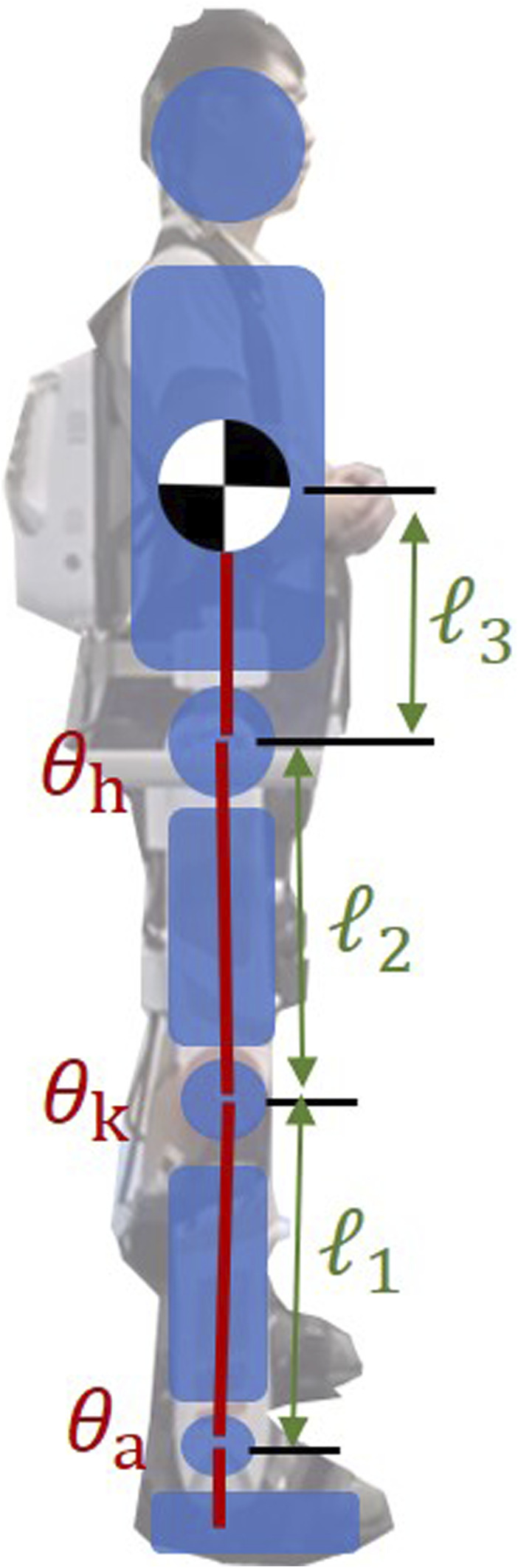
Virtual model for Human-exoskeleton system.

The integrated system of a human wearing an exoskeleton robot is effectively represented through a virtual model. This model, initially introduced by Pratt J. and colleagues (Pratt et al., 2001), serves as a computational framework within robotics and control systems to simulate dynamic interactions. It abstractly constructs components such as springs, dampers, and forces, which, although not physically present, are essential for mathematically simulating the system’s dynamics. To enhance understanding, Figure 1 now includes detailed annotations and vectors representing the equivalent torques at the hip, knee, and ankle joints, alongside a simplified diagram that maps these virtual components to their respective physical counterparts in the exoskeleton structure. This visualization aids in comprehending how virtual forces and torques are applied within the model to mimic real-world physical interactions between the user and the exoskeleton.

$$\left[\begin{array}{c} x\\ z\\ \theta \end{array}\right]=\left[\begin{array}{c} l_{1}S_{a}+l_{2}S_{ak}+l_{3}S_{\mathrm{akh}}\\ l_{1}C_{a}+l_{2}C_{ak}+l_{3}C_{\mathrm{akh}}\\ \theta_{1}+\theta_{2}+\theta_{3} \end{array}\right]$$ (1)

Here, *l*_1_, *l*_2_ and *l*_3_ stand for the lower leg’s length, the thigh’s length, and the distance between the hip joint and the body’s center of mass. The angles of the hip, knee, and ankle joints are denoted by *θ*_*h*_, *θ*_*k*_ and *θ*_*a*_ respectively. Additionally, the terms *S*_*ak*_ and *C*_*ak*_ represent the shorthand for sin(*θ*_*a*_ + *θ*_*k*_) and cos(*θ*_*a*_ + *θ*_*k*_).

Taking the derivative of Eq. 1 provides Eq. 2, the Jacobian matrix during a bipedal stance:

$$J=\left[\begin{array}{ccc} l_{1}C_{a}+l_{2}C_{ak}+l_{3}C_{\mathrm{akh}} & l_{2}C_{ak}+l_{3}C_{\mathrm{akh}} & l_{3}C_{\mathrm{akh}}\\ -l_{1}S_{a1}-l_{2}S_{ak}-l_{3}S_{\mathrm{akh}} & -l_{2}S_{ak}-l_{3}S_{\mathrm{akh}} & -l_{3}S_{\mathrm{akh}}\\ 1 & 1 & 1 \end{array}\right]$$ (2)

Using this Jacobian matrix, it is possible to compute the equivalent joint torque from the virtual force and torque at the center of mass, as shown in Eq. 3:

$$\left[\begin{array}{c} \tau_{a}\\ \tau_{k}\\ \tau_{h} \end{array}\right]=J^{T}\left[\begin{array}{c} F_{x}\\ F_{z}\\ \tau \end{array}\right]$$ (3)

In this context, the variables *τ*_*a*_, *τ*_*k*_, *τ*_*h*_ represent the torques exerted on the ankle, knee, and hip joints, respectively, while *F*_*x*_, *F*_*z*_ and *τ* denote the resultant force and torque acting upon the body’s center of mass. Upon expanding the equation, we arrive at the following Eq. 4:

$$\begin{array}{c} \tau_{a}=\left(l_{1}C_{a}+l_{2}C_{ak}+l_{3}C_{\mathrm{akh}}\right)F_{x}-\left(l_{1}S_{a1}+l_{2}S_{ak}+l_{3}S_{\mathrm{akh}}\right)F_{z}+\tau \\ \tau_{k}=\left(l_{2}C_{ak}+l_{3}C_{\mathrm{akh}}\right)F_{x}-\left(l_{2}S_{ak}+l_{3}S_{\mathrm{akh}}\right)F_{z}+\tau \\ \tau_{h}=l_{3}C_{\mathrm{akh}}F_{x}-l_{3}S_{\mathrm{akh}}F_{z}+\tau \end{array}$$ (4)

In the context of human standing, the knee remains unbent, thereby rendering the torque at the knee joint, denoted by *τ*_*k*_, equal to zero. Consequently, we deduce Eq. 5:

$$\tau =\left(l_{2}S_{ak}+l_{3}S_{\mathrm{akh}}\right)F_{x}-\left(l_{2}C_{ak}+l_{3}C_{\mathrm{akh}}\right)F_{z}$$ (5)

Upon substituting Eq. 4 into Eq. 3 with the condition *θ*_*k*_ = 0, and following a process of simplification and reorganization, we obtain the following Eq. 6:

$$\tau_{a}+\tau_{h}=\left(l_{1}+l_{2}\right)S_{a}F_{x}-\left(l_{2}+l_{2}\right)C_{a}+F_{z}$$ (6)

We observe that the forces acting on the Center of Mass (CoM), denoted as *F*_*x*_ and *F*_*z*_, are independent of the link length *l*_3_ and the angle *θ*_*h*_. For simplification purposes, the term (*l*_1_ + *l*_2_)*S*_*a*_ is represented as *z*, and (*l*_1_ + *l*_2_)*C*_*a*_ is represented as *x*.

Further assuming that the vertical distance between the CoM and the ground remains constant, denoted as *z* ≡ *z*_0_, and considering that $F_{x}=m\ddot{x}$ and *F*_*z*_ = *mg*, we derive the following Eq. 7:

$$\ddot{x}=\omega^{2}x+\frac{\tau_{a}}{z_{0}m}+\frac{\tau_{h}}{z_{0}m}$$ (7)

For an exoskeleton in a standing position, to prevent any slip between the exoskeleton footplate and the ground, it is crucial that the virtual force complies with the condition: *F*_*x*_/*F*_*z*_ < *μ*, where *μ* symbolizes the static friction coefficient of the surface. Concurrently, to ensure that both feet remain grounded, the virtual force should also adhere to the condition:*F*_*z*_ > 0.

### 2.2 Simplified model and orbital energy

**FIGURE 2:**
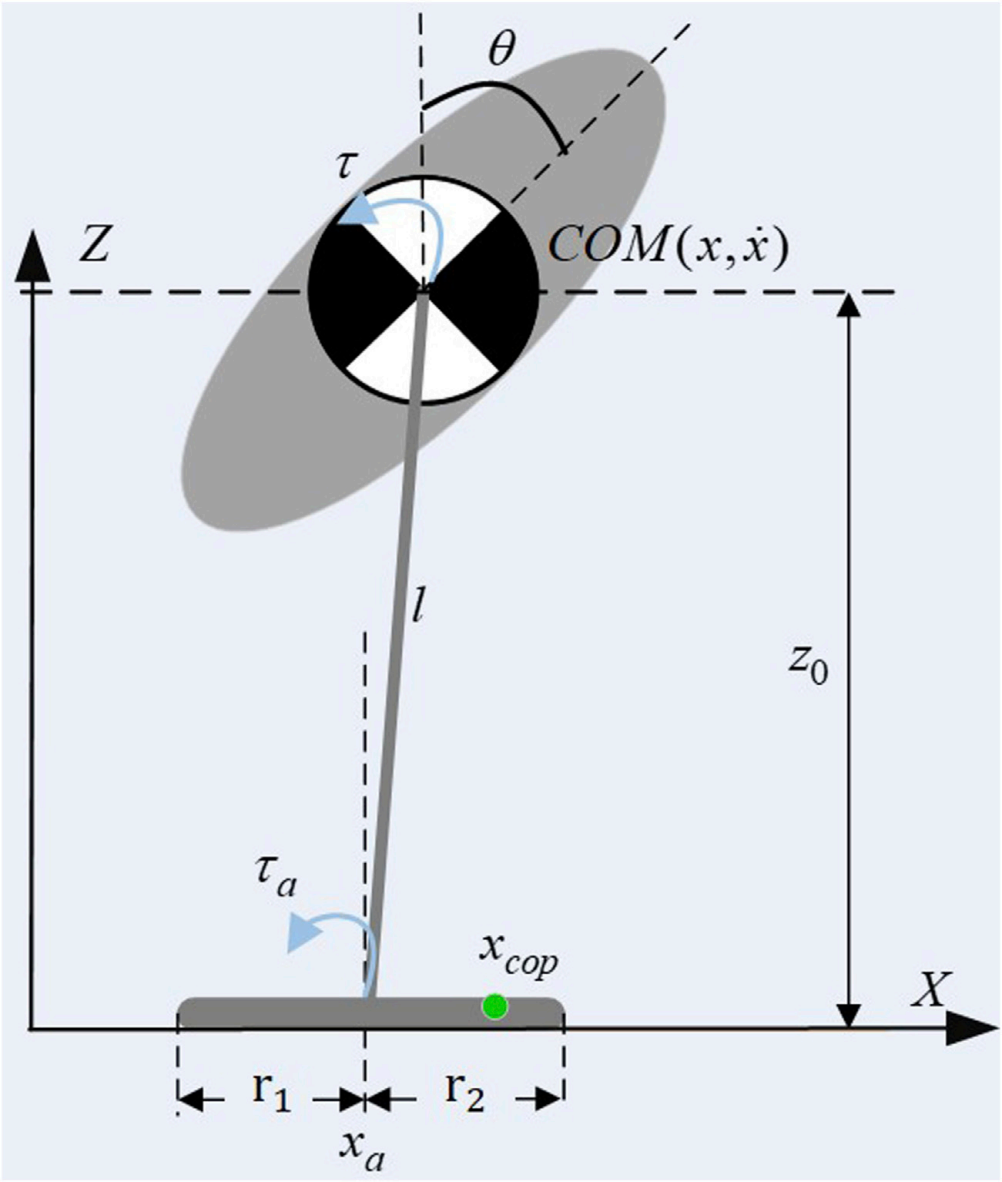
Flywheel inverted pendulum with finite feet.

Based on Eq. 7, and subject to certain constraints, the virtual model can be effectively simplified to a Flywheel Inverted Pendulum (FIP) Model. As depicted in Figure 2, this model consists of a scalable, massless link, denoted as *l*, a mass flywheel, which is controllable via a torque *τ*, and a foot with a finite length ranging from −*r*_1_ to *r*_2_. The combined center of mass of both the wearer and the exoskeleton system is represented by the center of this mass flywheel.

Pratt et al. (2006) conducted a detailed analysis of the balance recovery theory for the flywheel inverted pendulum model under disturbances, which can be articulated using the energy orbit expression by Kajita et al. (2010). The system can be conceptualized as a spring with unit mass and a stiffness of −*g*/*z*_0_. The orbital energy is expressed as the difference between kinetic and potential energy, as shown in Eq. 8:

$$E_{\mathrm{LIP}}=\frac{1}{2}{\dot{x}}^{2}-\frac{g}{2z_{0}}x^{2}$$ (8)

‘Capture Point’ refers to a specific point on the ground where, if the robot steps, it can achieve balance and stop without further movement. The system’s capture point can be determined when *E*_*LIP*_ = 0, leads us to the following Eq. 9:

$$x_{cp}=\dot{x}\sqrt{\frac{z_{0}}{g}}$$ (9)

where *z*_0_ represents the constant height of the overall center of mass of the human-machine system relative to the ground, *g* is the gravitational acceleration. The condition *E*_*LIP*_ = 0 leads to two solutions: $\dot{x}=\pm x\sqrt{g/z_{0}}$ . The negative solution $\dot{x}=-x\sqrt{g/z_{0}}$ indicates that without external forces, the system will naturally tend to return to the initial balanced state. In contrast, the positive solution $\dot{x}=x\sqrt{g/z_{0}}$ relates to the capture point, where the robot steps forward to a new equilibrium, stopping further motion.

**FIGURE 3:**
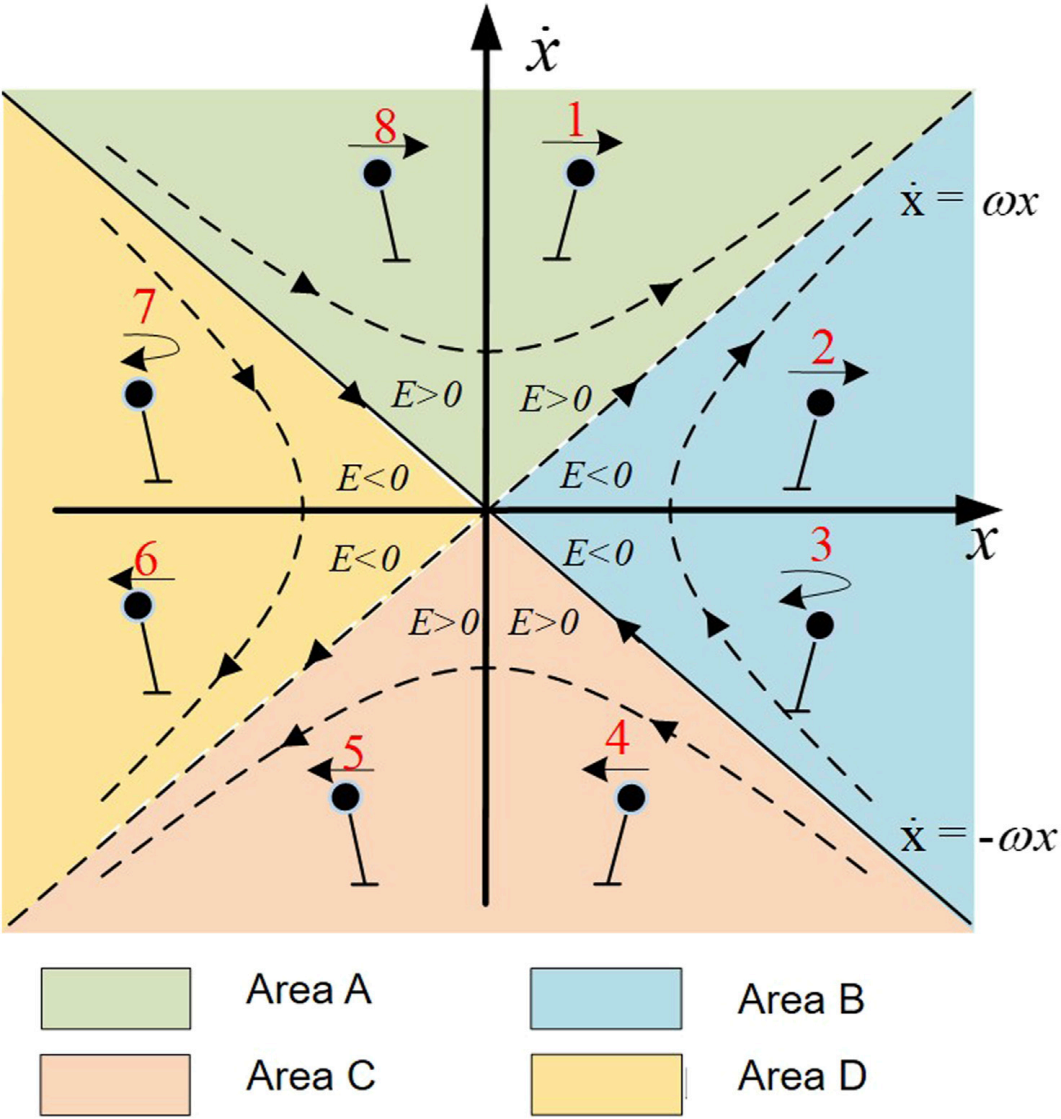
Diagram of orbital energy.

Figure 3 shows the phase diagram of a FIP, the straight line formed by the position and velocity of the center of mass is called a stable orbit. For a given state, FIP only has one capture point, and its state is transformed into a stable eigenvector.

In order to apply the CP balance evaluation index to exoskeleton robots, we propose the ‘Orbit Energy’ (OE) to evaluate the stability of the human-exoskeleton system. OE is defined in Eq. 10:

$$S=x+\frac{\dot{x}}{\omega }$$ (10)

By inputting the centroid position and velocity of the human-exoskeleton system, the current stable state of the system can be determined. When subjected to external disturbances, substituting the position *x* and velocity $\dot{x}$ into Eq. 10 yields an orbit energy.

For the FIP system, an OE threshold *Sth* exists. When the orbit offset caused by disturbances is less than this threshold, i.e., *S* < *Sth*, the system can autonomously recover stability.

## 3 Balance recovery strategy

Research on human balance mechanisms underscores the importance of the hip and ankle joints in regaining equilibrium after disturbances, as highlighted in studies like (Winter, 1995). Healthy individuals, despite having most of their body mass located far from the ground, can adeptly recover balance due to their flexible joints, developed muscles, and the cerebellum’s sophisticated motor control.

However, the engineering technologies, degrees of freedom, flexibility, and control systems in current lower limb exoskeleton robots do not fully match these human balance mechanisms. Insights gained from human balance recovery strategies, as identified in research such as Winter’s (Winter, 1995), are crucial. These strategies, specifically the ankle and ankle-hip strategies, are the focus of this paper, while the step strategy is considered out of scope.

### 3.1 Self-stabilization range

To better analyze the characteristics of FIP using EO, Eq. 8 can be reformulated into a state-space representation as Eq. 11:

$$\left[\begin{array}{c} \dot{x}\\ \ddot{x} \end{array}\right]=\left[\begin{array}{cc} 0 & 1\\ \omega^{2} & 0 \end{array}\right]\left[\begin{array}{c} x\\ \dot{x} \end{array}\right]+\left[\begin{array}{cc} 0 & 0\\ \frac{1}{z_{0}m} & \frac{1}{z_{0}m} \end{array}\right]\left[\begin{array}{c} \tau_{a}\\ \tau_{h} \end{array}\right]$$ (11)

In this context, the state vector is defined as $x={\left[\begin{array}{cc} x & \dot{x} \end{array}\right]}^{T}$ representing the position and velocity, while the torques applied by the ankle and hip joints are denoted by $u={\left[\begin{array}{cc} \tau_{a} & \tau_{h} \end{array}\right]}^{T}$ . The output is represented as Eq. 12:

$$y=\left[\begin{array}{cc} 1 & \frac{1}{\omega } \end{array}\right]\left[\begin{array}{c} x\\ \dot{x} \end{array}\right]$$ (12)

The output variable *y*(*k*) represents the capture point position, which is also the target variable we want to optimize and control. In the absence of joint torque assistance for balance, that is, when *τ*_*a*_ and *τ*_*h*_, we can derive the open-loop analytical solution of the state space Eq. 13:

$$\left[\begin{array}{c} x\\ \dot{x} \end{array}\right]=\left[\begin{array}{cc} cosh\left(\omega t\right) & \frac{1}{\omega }sinh\left(\omega t\right)\\ \omega sinh\left(\omega t\right) & cosh\left(\omega t\right) \end{array}\right]\left[\begin{array}{c} x_{0}\\ {\dot{x}}_{0} \end{array}\right]$$ (13)

The contact between the exoskeleton robot’s foot and the ground is not a single point but rather an area. When the capture point lies within this area, the system can autonomously regain balance without external assistance. This area is denoted as Eq. 14:

$$-r_{1}<x+\frac{\dot{x}}{\omega }<r_{2}$$ (14)

Where *r*_1_ and *r*_2_ denote the boundary of the support polygon. Substituting Eq. 13 into the stable constraint Eq. 14 yields Eq. 15:

$$\begin{array}{c} -r_{1}<x_{0}cosh\left(\omega t\right)+{\dot{x}}_{0}\frac{1}{\omega }sinh\left(\omega t\right)\\ +x_{0}sinh\left(\omega t\right)+{\dot{x}}_{0}\frac{1}{\omega }cosh\left(\omega t\right)<r_{2} \end{array}$$ (15)

When the system’s center of mass, represented by *x*, meets the aforementioned criteria, the system can autonomously regain stability; however, if these conditions are not met, the ankle strategy must be implemented to avert a fall.

### 3.2 Ankle strategy

The ankle strategy employs torque around the ankle joint to restore balance, particularly effective against minor disturbances by increasing muscle stiffness near the ankle. Drawing from this reflex mechanism, we counteract small external forces on the human-exoskeleton system by applying reverse torque at the exoskeleton’s ankle joint, while other joints remain stationary.

The most impactful torque profile on balance is achieved by applying the maximum possible acceleration to the flywheel in a single direction, followed by a deceleration phase that halts the flywheel at its furthest angular position. The expression for the torque is Eq. 16:

$$\tau \left(t\right)=\tau_{\max}u\left(t\right)-2\tau_{\max}u\left(t-T_{R1}\right)+\tau_{\max}u\left(t-T_{R2}\right)$$ (16)

The torque at any given time *t*, denoted by *τ*(*t*), is modeled as starting from a base value determined by the maximum torque *τ*_max_ that the joint is capable of exerting. This initial torque is then modified by a unit step function initiated at time T, which represents the onset of the torque application.

The torque undergoes a reduction, specifically a subtraction of twice the maximum torque, beginning at time *T*_*R*1_ which marks the transition from acceleration to deceleration of the flywheel. Finally, an additional instance of the maximum torque is factored in at time *T*_*R*2_, reflecting the moment when the flywheel ceases movement entirely.

At this juncture, the orbit energy (OE) is equivalent to *x*_*cp*_, necessitating an analysis of the system’s zero-state and zero-input responses, followed by the simultaneous solution of equations. Pratt has already conducted a detailed derivation of this, which can be referenced in the paper (Pratt et al., 2006). The calculation result is presented as Eq. 17:

$$x_{cp}=-\frac{1}{\omega }\left(-{\dot{x}}_{0}+\frac{\tau_{a,max}}{mg}\left[\frac{e^{\omega T_{R2}}-2e^{\omega \left(T_{R2}-T_{R1}\right)}+1}{e^{\omega T_{R2}}}\right]\right)$$ (17)

The position at which the system can be considered captured is given by the negative of *x*_0_. To determine the opposite limit of the Capture Region, one may replicate the process using the minimum torque *τ*_min_.

If the objective is to ascertain a Capture Point devoid of angular momentum influence, the procedure is the same except that *T*_*R*1_ is set to zero. The duration *T*_*R*2_ should be sufficient to halt any ongoing rotational motion of the flywheel, and thus, the value of *x*_0_ can be calculated in the same manner as previously described.

Based on the calculated capture point *x*_*cp*_ position from the equation, if it falls within the support region (i.e., the foot area, between −*r*_1_ and *r*_2_), the ankle torque strategy is used for balance recovery; if beyond that range, the strategy of applying torque simultaneously at the ankle and hip joints is needed.

### 3.3 Ankle-hip strategy

The ankle-hip strategy, which involves applying torque simultaneously to both the ankle and hip joints to restore balance (Winter, 1995), follows a calculation method similar to that of the capture point in the ankle strategy. The derivation here is omitted for brevity, with the resulting expression presented as Eq. 18:

$$x_{cp}=-\frac{1}{\omega }\left(-{\dot{x}}_{0}+\frac{\tau_{a,max}+\tau_{h,max}}{mg}\left[\frac{e^{\omega T_{R2}}-2e^{\omega \left(T_{R2}-T_{R1}\right)}+1}{e^{\omega T_{R2}}}\right]\right)$$ (18)

The calculations based on the above equation indicate that when *x*_*cp*_ falls within the support polygon, the range between −*r*_1_ and *r*_2_, the ankle-hip strategy can be employed.

### 3.4 Model predictive controller

MPC, often referred to as receding horizon predictive control, is employed here in its discrete-time variant, which is executed through the employment of discrete-time state space functions. Within this framework, the discrete-time state space equation is expressed as a recursive relation, as shown in Eqs 19, 20, where the state vector at any subsequent instant is determined by the present state and control inputs.

$$x\left(k+1\right)=Ax\left(k\right)+Bu\left(k\right)$$ (19)

$$y\left(k\right)=Cx\left(k\right)$$ (20)

The system’s dynamic behavior is characterized by a linear relationship defined by a state matrix A, a control matrix B and a output matrix C.

The control objective is to regulate the capture point within the support area, ensuring that the final state of the system converges, with the position returning to the origin and velocity reaching zero. Therefore, we transfer the original state space equation into an augmented Eq. 21:

$$\left[\begin{array}{c} \delta x\left(k+1\right)\\ y\left(k+1\right) \end{array}\right]=\left[\begin{array}{cc} A & 0\\ CA & 1 \end{array}\right]\left[\begin{array}{c} \delta x\left(k\right)\\ y\left(k\right) \end{array}\right]+\left[\begin{array}{c} B\\ CB \end{array}\right]\delta u\left(k\right)$$ (21)

In this context, the augmented state vector is defined as $x={\left[\begin{array}{cc} \delta x & y \end{array}\right]}^{T}$ representing the state of the system and capture point. Consequently, the augmented output vector encompasses both the system state and the system output. This integration facilitates the application towards control objectives, as illustrated in Eq. 22:

$$y\left(k\right)=\left[\begin{array}{cc} 0 & 1 \end{array}\right]\left[\begin{array}{c} \delta x\left(k\right)\\ y\left(k\right) \end{array}\right]$$ (22)

To effectively implement MPC under the constraint of boundary conditions, it is imperative to define a cost function and constraints across a defined finite horizon.

$$\begin{array}{cccc} & \underset{u}{\text{minimize}} & & J=\overset{N}{\underset{k=0}{\sum }}y{\left(k\right)}^{T}Qy\left(k\right)+\delta u{\left(k\right)}^{T}R\delta u\left(k\right)\\ & \text{subject to} & & -r_{1}\leq y\left(k\right)\leq r_{2},\;k=1,\ldots ,N,\\ & & & \tau_{\text{min}}\leq u\left(k\right)\leq \tau_{\text{max}},\;k=1,\ldots ,N,\\ & & & \theta_{\text{min}}\leq \theta_{a}\left(k\right)\leq \theta_{\text{max}},\;k=1,\ldots ,N,\\ & & & \theta_{\text{min}}\leq \theta_{h}\left(k\right)\leq \theta_{\text{max}},\;k=1,\ldots ,N. \end{array}$$ (23)

This cost function, Eq. 23, is composed of the cumulative stage costs. Each stage cost is a quadratic function of the state and control input vectors, which are weighted by the matrices Q and R, respectively.

$$\tau_{\mathrm{opt}}=\left[\tau_{\mathrm{opt}}\left(0\right),\tau_{\mathrm{opt}}\left(1\right),\ldots ,\tau_{\mathrm{opt}}\left(N-1\right)\right]$$ (24)

$$x_{\mathrm{opt}}=\left[x_{\mathrm{opt}}\left(1\right),x_{\mathrm{opt}}\left(2\right),\ldots ,x_{\mathrm{opt}}\left(N\right)\right]$$ (25)

Solved via an optimization solver, Eq. 24 and 25, the optimal control sequence *τ*_*opt*_ and *x*_*opt*_ are obtained. This includes the initial control signal *τ*_*opt*_(0) applied to the system, consequently generating the actual state *x*(1).

These actual states, *x*(*k*), are measured and may align with or differ from the predicted states *x*_*opt*_(*k*). At the next time step, these actual states serve as the new starting point for the subsequent optimization problem, occurring at the sample time k. This process is cyclically repeated by the MPC, which consistently recalibrates the control inputs for the system based on the latest observed state. This leads to a continuous observation and adjustment cycle, making the MPC a recursive algorithm for achieving optimal control.

## 4 Simulation verification

### 4.1 Simulation environment

**FIGURE 4:**
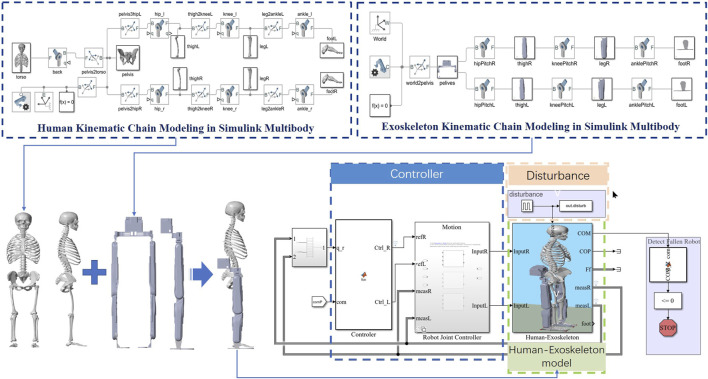
Simulation science and MPC control frame.

In order to verify the effectiveness of the balance recovery control strategy, provide reference data for human-exoskeleton experiments, and ensure the safe and orderly conduct of human-exoskeleton experiments, we built a human-machine system model in Matlab Simulink SimMechanics and conducted simulation verification. The lower limb exoskeleton model is modeled using Solidworks and the model parameters are imported into SimMechanics, as shown in Figure 4.

The exoskeleton robot has a mass of 26 kg, with each leg equipped with hip joint flexion/extension (HFE), hip abduction/adduction (HAA), hip medial and lateral rotation (HMR), knee joint flexion/extension (KFE), ankle joint dorsiflexion/plantar flexion (DF/PF), and ankle inversion/eversion (AIE), totaling 6 degrees of freedom per leg.

### 4.2 Simulation setup

The mass of the human body model is 75 kg, the mass of the exoskeleton model is 26 kg, and the length of the exoskeleton sole plate is 0.26 m (the connection between the ankle joint and the sole of the foot is in the forward and backward direction). The stiffness of ADP is set to 130 Nm, the damping is set to 5, and the maximum torque is set to 70Nm; The stiffness of HFE and KFE is set to 1000N, and the damping is set to 10; Prohibit movement of HML, HAA, AEI.

Simulation 1: We applied an ankle strategy for balance recovery in an exoskeleton, inducing pulse disturbances ranging from 0 to 500 N on the backpack. Each disturbance was 0.5 s long with a 10-s interval. Disturbances increased by 10 N increments, over 101 simulations. No additional countermeasures were used if the strategy failed.

Simulation 2: The ankle-hip strategy was tested with disturbances from 0 to 600N, also in 10 N increments and with the same duration and interval as Simulation 1. This series included 121 simulations, with no extra measures for failed recoveries.

### 4.3 Simulation result

**FIGURE 5:**
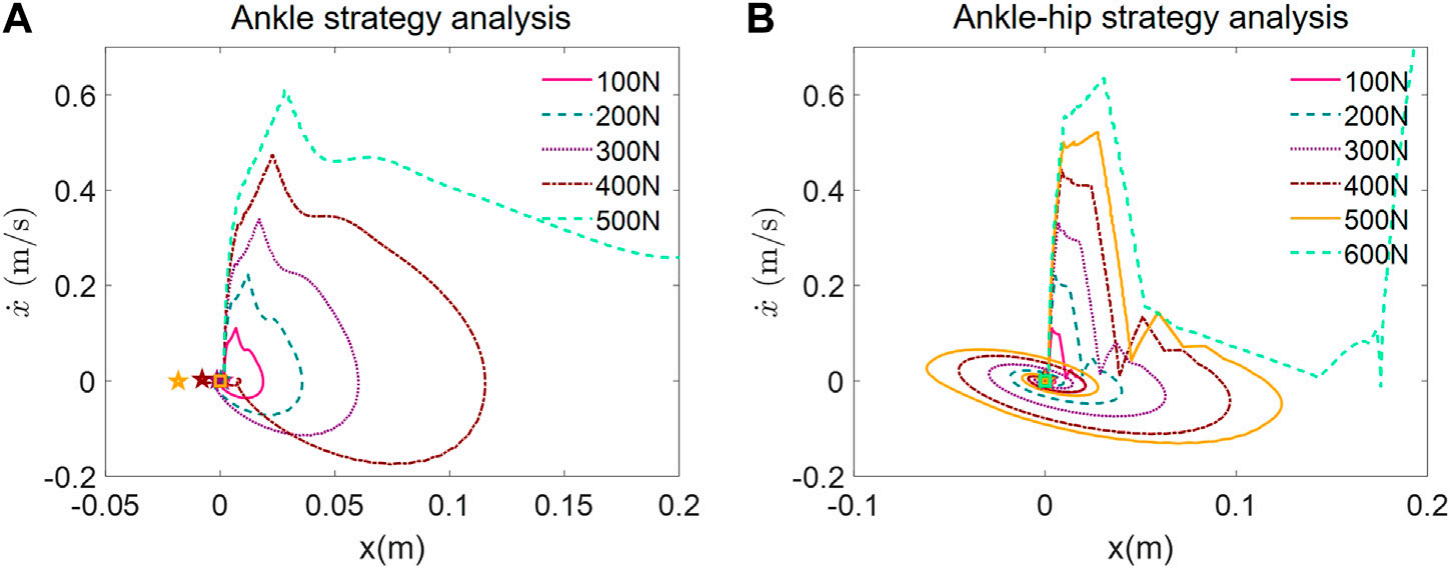
Phase portrait of push recovery simulation. **(A)** Ankle strategy analysis. **(B)** Ankle-hip strategy analysis.

Ankle Strategy Analysis: Under the ankle strategy, as shown in Figure 5A, the CoM trajectories exhibit a trend of increasing displacement with higher magnitudes of applied force. The phase plots reveal that for lower disturbances (up to 100 N), the CoM trajectories form tight, closed loops around the origin, indicating effective balance recovery and stability. As the disturbance force increases, the loops become larger and more elongated, suggesting that the ability of the ankle strategy to maintain balance diminishes with greater perturbations.

The trajectories for the highest forces show significant deviation from the origin, indicating that the ankle strategy may not be sufficient to counteract higher levels of disturbance. The absence of additional recovery measures upon strategy failure suggests the importance of implementing multi-joint strategies in exoskeletons for more effective balance recovery.

Ankle-Hip Strategy Analysis: With the implementation of the ankle-hip strategy, as shown in Figure 5B, the CoM trajectories demonstrate a more complex pattern. For disturbances up to 400N, the phase portraits show closed loops, although they appear to be more spread out compared to the ankle strategy, implying a more active and potentially more controlled recovery process. Notably, at disturbances of 500 N and above, the trajectories start to exhibit open loops, indicating instances of failure to recover balance.

The inclusion of the hip strategy appears to enhance the balance recovery capability of the exoskeleton, as evidenced by the ability to withstand higher disturbances before strategy failure. However, similar to the ankle strategy, the ankle-hip strategy also reaches a threshold beyond which it cannot maintain balance, as seen with the 600 N disturbance.

## 5 Experiment verification

To validate the devised standing balance recovery method for the human-exoskeleton system, we conducted a disturbance experiment utilizing a lower limb exoskeleton robot.

### 5.1 Milebot exoskeleton BEAR-H1

**FIGURE 6:**
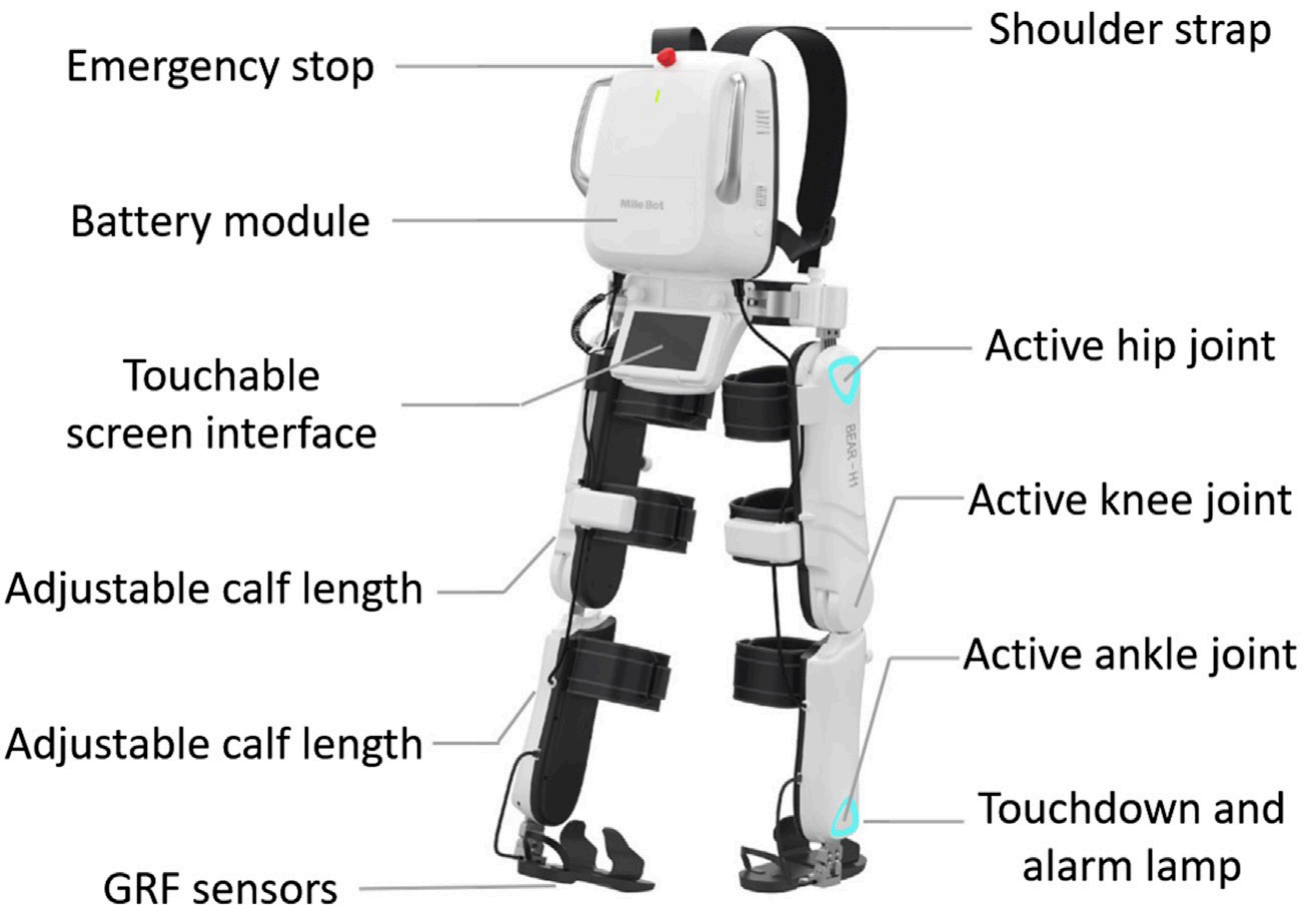
Milebot exoskeleton BEAR-H1.

BEAR-H1, as shown in Figure 6, is a wearable, battery-powered lower-limb exoskeleton developed by Shenzhen Milebot company with the purpose of assisting in gait rehabilitation training. The specification of BEAR-H1 is shown in Table 1. The BEAR-H1 features three actively compliant motor-actuated joints on each leg, facilitating rotations along the hip joint, knee joint, and ankle joint within the sagittal plane. The length of the thigh and calf is adjustable to accommodate individuals with heights ranging from 150 to 190 cm and weighing less than 85 kg. To monitor gait, a touchable screen is integrated beneath the back panel, and a ground reaction force (GRF) sensor is embedded in the sole to detect touchdown events.

**TABLE 1:** Specification of Milebot BEAR-H1.

Parts	Parameters
Input Power	21.6 V, 5 A
Equipment mass	26 kg
Maximum patient weight	85 kg
Applicable height range	155 ∼ 190 cm
Waist width	290 ∼ 420 mm
Thigh length	360 ∼ 480 mm
Calf length	345 ∼ 450 mm
Signal acquisition frequency	100 Hz

### 5.2 Exoskeleton control framework

**FIGURE 7:**
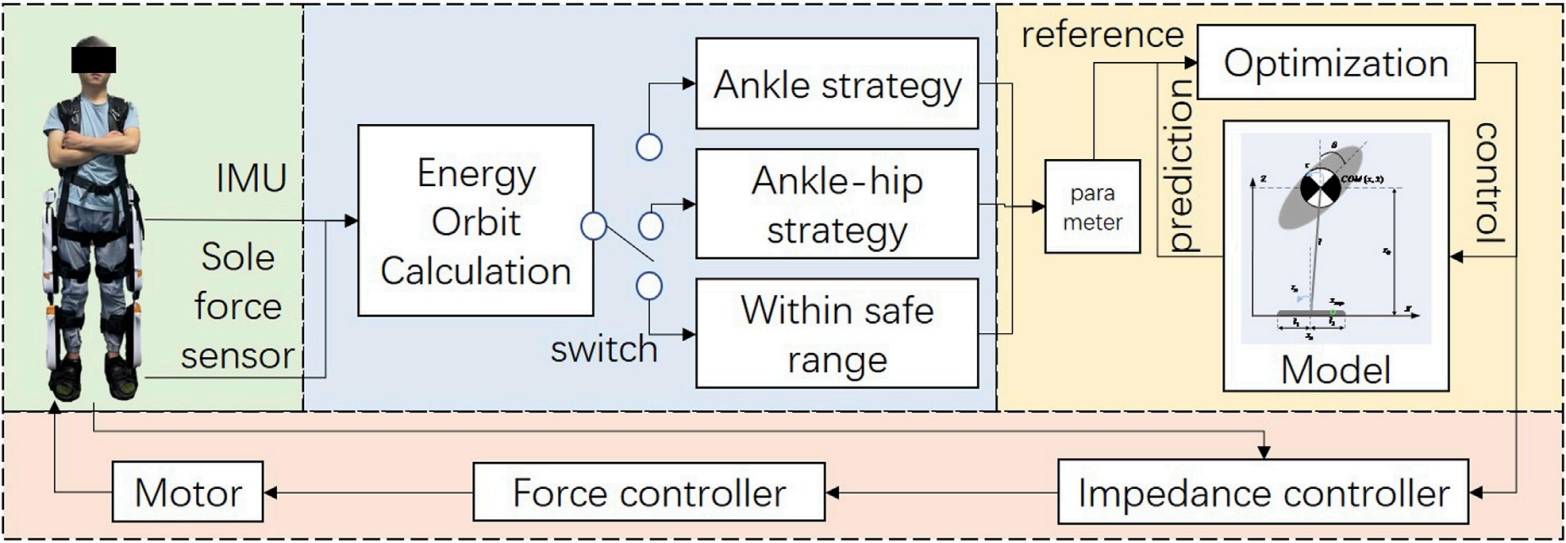
Control framework of human-exoskeleton system.

The control framework is shown in Figure 7. The depicted control framework for an exoskeleton employs real-time data from an Inertial Measurement Unit (IMU) and sole force sensors to calculate the dynamic state of the system. The system dynamically toggles between ankle and ankle-hip balance strategies, selecting the optimal approach based on the calculated energy state and predefined safety thresholds.

The selected strategy informs an advanced optimization routine within a predictive model that anticipates the exoskeleton’s future trajectory, enabling the calculation of ideal control inputs. These inputs modulate the force controller, which drives the motor, and the impedance controller, which fine-tunes the exoskeleton’s responsiveness, ensuring the system’s stability and congruent assistance with the user’s standing.

### 5.3 Experiment setup

Before the experiment started, the participants put on the exoskeleton, and electrodes were affixed to their leg skin for the collection of surface electromyography (sEMG) data. To ensure the safety of the experimental participants, a safety harness was suspended above the treadmill to prevent any unforeseen accidents.

Before each experiment session, participants were explicitly instructed not to engage in proactive balance recovery measures in response to external disturbances. However, it is worth noting that participants with intact limb motor function might exhibit conditioned reflexes leading to the spontaneous adoption of balance recovery strategies.

Research findings have established that the amplitude of sEMG signals reflects the degree of muscle activation and can indirectly indicate muscle strength. To assess the extent of the human body’s involvement in the balance recovery process, this study employed EMG acquisition sensors manufactured by NORAXON Company. These sensors were strategically placed on the participant’s tibialis anterior (TA), semitendinosus (ST), lateral gastrocnemius muscle (LG), peroneus longus (PL), rectus femoris (RF), vastus medialis (VM), vastus lateralis (VL), and biceps femoris muscle (BF), resulting in a total of 16 channels for sEMG data collection and subsequent calculation of muscle activation.

For this experiment, a group of two healthy male volunteers was selected, with average ages, heights, and weights of 25 ± 2 years old, 1.74 ± 0.08 m, and 69 ± 10.9 kg, respectively. Importantly, none of the participants had a history of neurological disorders.

Experiment 1: Each joint motor of the exoskeleton is configured in zero-torque mode. After each volunteer puts on the exoskeleton, they are given a 3-min period to become acquainted with it. Subsequently, the experiment commences by applying a horizontal forward thrust of 100 N at the position of the exoskeleton backpack. Each thrust lasts approximately 500 milliseconds, with a total of 10 repetitions, and a 5-s interval between each push. Data from the electromyography sensors and the exoskeleton sensors are recorded. The aforementioned procedure is then repeated, but this time with a horizontal backward pulling force of 100 N at the backpack.

Experiment 2: Configure the exoskeleton in ankle strategy mode and replicate the steps outlined in Experiment 1.

Experiment 3: Configure the exoskeleton in ankle-hip strategy mode and replicate the steps outlined in Experiment 1.

### 5.4 Experiment result and analysis

**FIGURE 8:**
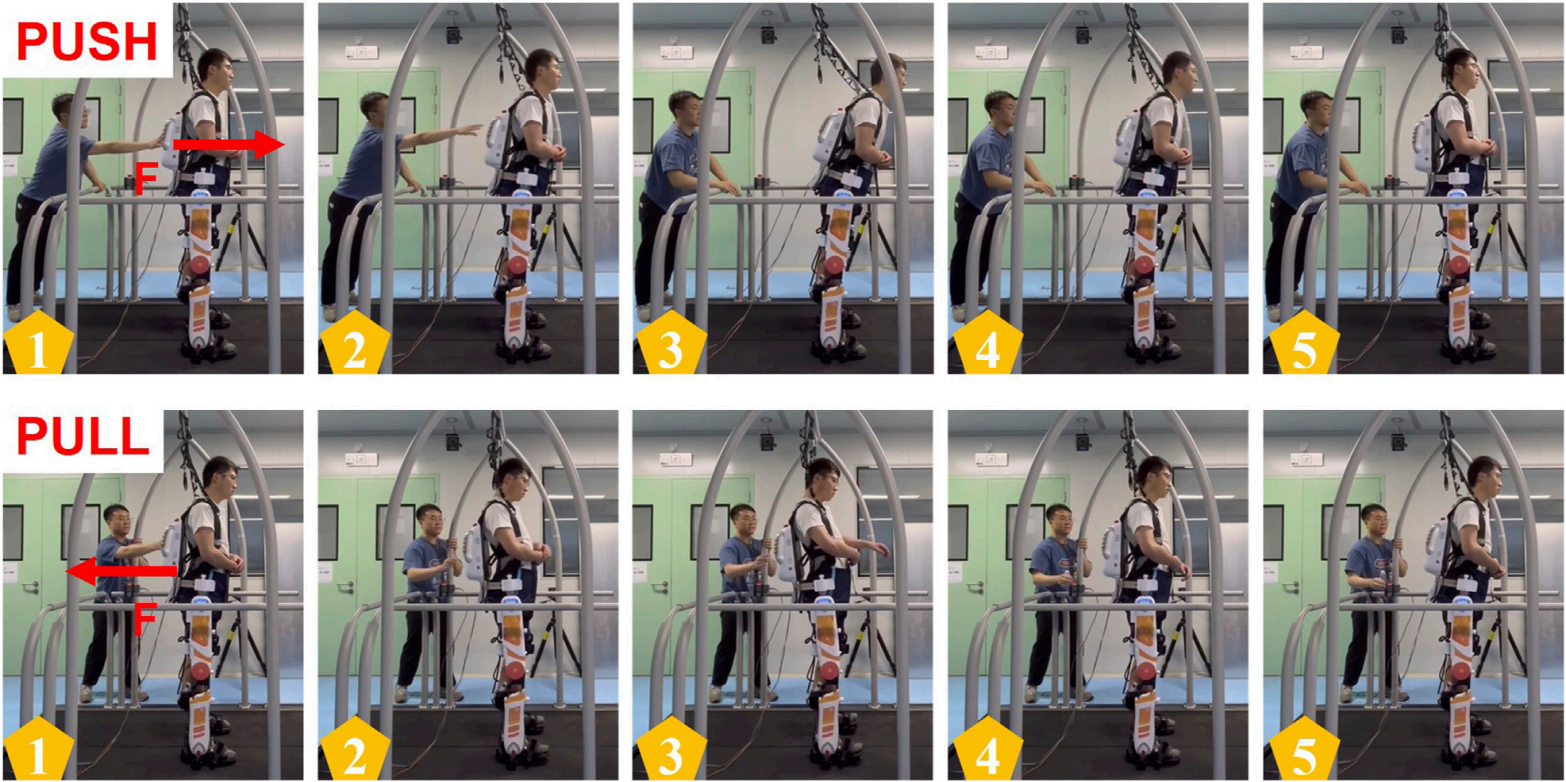
Experiment scene of push and pull recovery experiment.

The experiment scene is shown in Figure 8. The exoskeleton’s sensor data is acquired at a frequency of 100Hz, while the sEMG data is sampled at 2000 Hz. The sEMG signal collected by the EMG sensor often contains substantial noise. According to prior research (Chu et al., 2006), the typical frequency range of the sEMG signal falls between 0 and 500 Hz.

To mitigate the noise present in the EMG signal, we applied pre-processing techniques to the surface sEMG data. This involved a 25 Hz fifth-order Butterworth high-pass filter and rectification, followed by a 5 Hz fifth-order Butterworth low-pass filter, as well as data normalization, as described in previous work (Zhang et al., 2020). The resulting normalized signal was employed for neural activation analysis, utilizing the following Eq. 26:

$$u_{j}\left(t\right)=\alpha e_{j}\left(t-d\right)-\beta_{1}u_{j}\left(t-1\right)-\beta_{2}u_{j}\left(t-2\right)$$ (26)

Where *β*_1_ = *C*_1_ + *C*_2_, *B*_2_ = *C*_1_ ⋅ *C*_2_, and *α* = *β*_1_ + *β*_2_ + 1. The muscle activation a(t) can be denoted as Eq. 27:

$$a_{i}\left(t\right)=\frac{e^{A_{i}u_{i}\left(t\right)}-1}{e^{A_{I}}-1}$$ (27)

Where A is a nonlinear shape coefficient, representing the degree of nonlinearity between nerve activation intensity and muscle activation intensity, and the value range of A is [-3,0] (Mantoan et al., 2015).

#### 5.4.1 Analysis of joint angle responses in exoskeleton-assisted push recovery experiment

**FIGURE 9:**
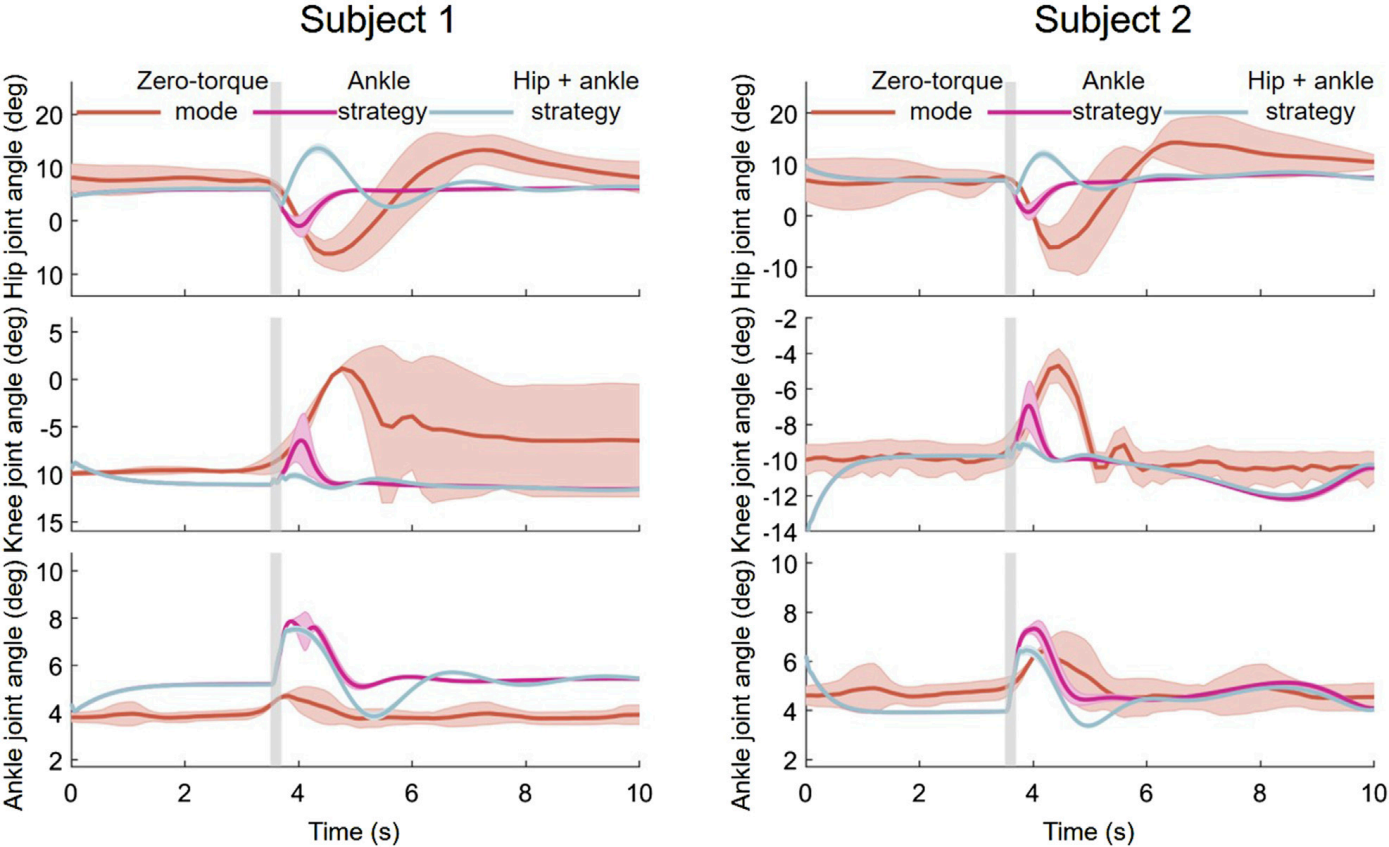
Joint angle responses during push recovery experiment.

Figure 9 depicts data from push recovery Experiment, showing the hip, knee and ankle joint angles over time in response to the controlled disturbance. The Figure shows three distinct phases for each joint: Zero-torque mode, Ankle strategy, and Ankle-hip strategy. These phases are likely indicative of different configurations of the exoskeleton used during the experiments.

Zero-torque Mode Analysis: In the zero-torque mode, the exoskeleton does not provide active force assistance, and changes in joint angles are primarily controlled by the subject’s autonomous movements. The experimental results indicate that after disturbances, both the hip and ankle joints exhibit certain fluctuations in angles, reflecting the subjects’ reliance on their body’s innate strategies to regain balance in the absence of exoskeleton assistance.

Ankle Strategy Analysis: When switched to the ankle strategy, the exoskeleton actively adjusts the ankle joint angles to counteract disturbances. In this experiment, the ankle joint’s response is more pronounced, suggesting that the ankle strategy plays a key role in maintaining and restoring balance. The hip and knee joints respond less but still make slight adjustments to aid in balancing.

Ankle-Hip Strategy Analysis: In the hip-ankle strategy, the exoskeleton concurrently adjusts both the hip and ankle joints to respond more comprehensively to disturbances. The figures show that the hip joint undergoes more significant dynamic changes, indicating that under this strategy, both the hip and ankle joints work together, providing a more complex mechanism for balance recovery. The knee joint’s angle fluctuations also increase, which may accommodate the larger range of motion from the hip and ankle joints.

#### 5.4.2 Analysis of sEMG in exoskeleton-assisted push recovery experiment

**FIGURE 10:**
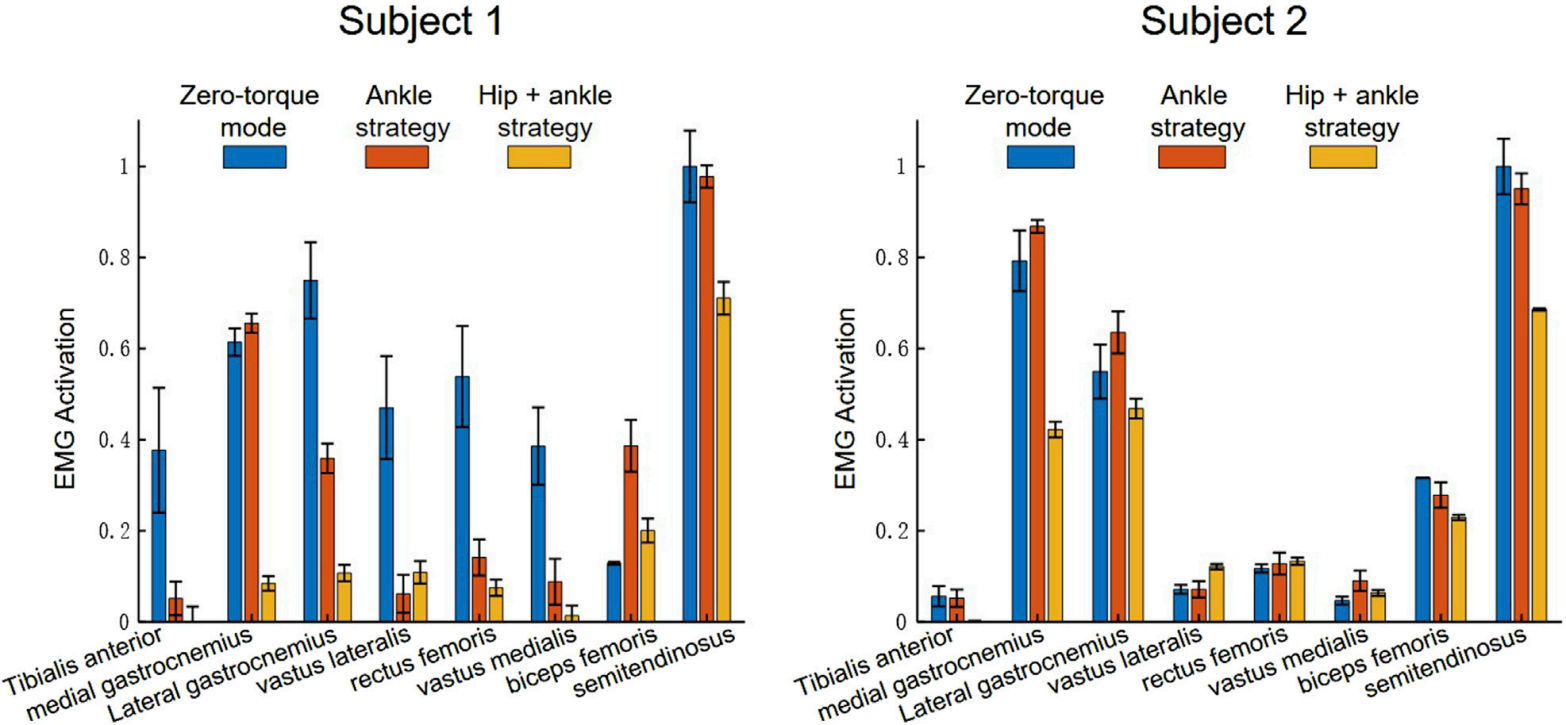
sEMG activation during push recovery experiment.

The sEMG results depicted in Figure 10 shows the mean and standard deviation of the lower limb muscle activation of the wearer under 10 perturbations in the process of forward and backward pushing, and provides a comparative quantification of muscle activations across three modes of exoskeleton operation, offering insights into the biomechanical implications of each strategy.

Zero-Torque Mode Analysis: In the Zero-torque mode, where the exoskeleton operates without providing active assistance to the wearer, the sEMG data indicates a baseline level of muscle activation. This mode reflects the user’s natural muscular response to the perturbations, with moderate activation across all muscle groups. The gastrocnemius muscles display relatively higher activation, which may suggest a natural inclination to utilize the ankle strategy even without exoskeleton assistance.

Ankle Strategy Mode Analysis: Transitioning to the Ankle strategy, there is an apparent shift in the activation pattern. The medial and lateral gastrocnemius muscles exhibit a notable increase in activation, reinforcing their role in the ankle strategy’s balance recovery mechanism. This increase suggests that the exoskeleton’s ankle strategy enhances the natural response by specifically augmenting the force production in these muscles to stabilize the user following a perturbation.

Ankle-Hip Strategy Mode Analysis: The Hip-ankle strategy mode demonstrates a distributed pattern of muscle activation, with the semitendinosus, and biceps femoris muscles showing significant engagement. This indicates that the hip strategy, when combined with the ankle strategy, does not solely rely on the lower leg muscles but also engages the thigh muscles, likely providing a more robust and comprehensive balance recovery response.

#### 5.4.3 Analysis of muscle activation variability in exoskeleton-assisted push recovery experiment

As shown in Table 2 and Table 3, the mean sEMG activation levels and their associated standard deviations across multiple muscles provide insights into the exoskeleton’s effect on muscle engagement.

**TABLE 2:** Mean and standard deviation of sEMG activation—subject 1.

	Zero-torque mode		Ankle strategy		Hip-ankle strategy	
Muscle	Mean	SD (%)	Mean	SD (%)	Mean	SD (%)
TA	0.06	2.23%	0.05	1.91%	0.00	0.23%
MG	0.79	6.68%	0.87	1.42%	0.42	1.73%
LG	0.55	5.92%	0.64	4.63%	0.47	2.17%
VL	0.07	1.00%	0.07	1.80%	0.12	0.59%
RF	0.12	0.93%	0.13	2.39%	0.13	0.79%
VM	0.05	0.87%	0.09	2.24%	0.06	0.65%
BF	0.32	0.03%	0.28	2.76%	0.23	0.58%
ST	1.00	6.06%	0.95	3.39%	0.69	0.22%

**TABLE 3:** Mean and standard deviation of sEMG activation—subject 2.

	Zero-torque mode		Ankle strategy		Hip-ankle strategy	
Muscle	Mean	SD (%)	Mean	SD (%)	Mean	SD (%)
TA	0.38	13.71%	0.05	3.68%	0.00	3.38%
MG	0.61	2.99%	0.66	2.09%	0.08	1.60%
LG	0.75	8.37%	0.36	3.25%	0.11	1.80%
VL	0.47	11.31%	0.06	4.17%	0.11	2.47%
RF	0.54	11.09%	0.14	3.95%	0.08	1.78%
VM	0.39	8.50%	0.09	5.05%	0.01	2.20%
BF	0.13	0.31%	0.39	5.70%	0.20	2.63%
ST	1.00	7.86%	0.98	2.45%	0.71	3.59%

Muscle Activation Patterns: Across the operational modes, the gastrocnemius muscles (medial gastrocnemius, MG, and lateral gastrocnemius, LG) and the semitendinosus (ST) typically exhibit higher mean activations, indicating their significant role in balance and locomotion tasks. Notably, the mean activation levels for these muscles decrease from Zero-Torque Mode to the Ankle and Hip-Ankle Strategies, suggesting that active assistance by the exoskeleton reduces the muscular effort required by the user. The Ankle Strategy mode often shows a slight increase in muscle activation compared to the Hip-Ankle Strategy, which may reflect the specific demand placed on the ankle muscles to stabilize the posture when the hip is less engaged.

Variability in Activation: The standard deviation percentages reflect the variability in muscle activation within each mode. Generally, a high standard deviation indicates a larger variability in muscle response, which could be attributed to individual differences in muscle control, fatigue levels, or the consistency of the exoskeleton’s assistance. A lower standard deviation in the Ankle-Hip Strategy suggests that this mode offers a more consistent level of support, potentially leading to a more predictable and uniform response across different movements and perturbations.

Efficiency of Exoskeleton Assistance: The efficiency of exoskeleton assistance can be inferred from the reduction in mean activation levels from Zero-Torque Mode to the Ankle and Hip-Ankle Strategies. The data indicates that the active control strategies of the exoskeleton likely contribute to a more economical muscle activation, thereby conserving energy for the user.

#### 5.4.4 Summary and discussion

Across all experiments, the mean trajectories of joint angles and their surrounding shaded areas, indicating variability or confidence intervals, provide insight into the consistency of responses among different individuals or repeated trials. In the Ankle and Ankle-Hip strategies, the proximity of the shaded area to the mean trajectory line denotes a higher uniformity in subjects’ responses to perturbations, with a reduced variability. These outcomes suggest the efficacy of the “Orbit Energy” metric in conjunction with the MPC controller in dynamically modulating the balance of the human-exoskeleton system, ensuring precise torque control.

The sEMG analysis provides a clear depiction of muscle activation trends across various strategies. The data illustrate a notable refinement in activation patterns and a decrease in the dispersion of muscle responses when employing the Ankle and Ankle-Hip strategies. These strategies, informed by the “Orbit Energy” metric and regulated by the MPC controller, contribute to a more consistent and targeted approach to balance recovery. The resulting reduction in muscle activation variability not only underscores the precision of our control system but also implies a potential decrease in muscular metabolic demand during balance maintenance.

When applied to a wide range of users with different physical characteristics, the current framework presents scalability challenges. This diversity necessitates further research into adaptive algorithms capable of customizing balance recovery strategies to individual user profiles. Additionally, the current study’s scope limited the types of disturbances tested; future work should explore the system’s responsiveness to a wider range of unpredictable real-world scenarios.

## 6 Conclusion

In this paper, we have presented a control framework for standing balance recovery in lower limb exoskeleton robots. The key innovation lies in using the proposed ‘Orbit Energy’ (OE) metric to assess balance and trigger appropriate strategies. The OE integrates the position and velocity of the overall center of mass of the human-exoskeleton system. It allows the determination of stable states after disturbances, providing an effective basis for strategy selection. The ankle torque controller recovers balance against minor perturbations. For larger disturbances, the ankle-hip torque controller expands the recovery range. The model predictive control optimizes torque inputs to regulate the capture point within the base of support. Simulations conducted in Simulink verify that the OE threshold successfully distinguishes the self-recovery range from cases needing control assistance. Experiments with human subjects further validate the framework’s ability to reduce muscle effort in maintaining balance. This research underscores the significance and innovations of the proposed Orbit Energy metrics, marking a pivotal advancement in managing standing balance control for lower limb exoskeletons.

Our current approach primarily focuses on standing balance, without extending to the complexities of walking balance. Recognizing this limitation, our future research will delve into the fundamental principles of human locomotive balance control. By integrating these principles with the unique characteristics of the human-exoskeleton interface, we aim to develop a comprehensive balance control algorithm tailored for both standing and walking scenarios. This advancement will bridge the current gap in our methodology, offering a more holistic approach to balance management in lower limb exoskeletons.

## Data Availability

The raw data supporting the conclusions of this article will be made available by the authors, without undue reservation.

## References

[B1] Afschrift M. van Asseldonk E. van Mierlo M. Bayon C. Keemink A. Van Der Kooij H. (2022). Assisting walking balance using a bio-inspired exoskeleton controller. bioRxiv, 2022–2110. 10.1186/s12984-023-01205-9 PMC10303867 37370175

[B2] Baud R. Manzoori A. R. Ijspeert A. Bouri M. (2021). Review of control strategies for lower-limb exoskeletons to assist gait. J. NeuroEngineering Rehabilitation 18, 119–134. 10.1186/s12984-021-00906-3 PMC8314580 34315499

[B3] Beck O. N. Shepherd M. K. Rastogi R. Martino G. Ting L. H. Sawicki G. S. (2023). Exoskeletons need to react faster than physiological responses to improve standing balance. Sci. robotics 8, eadf1080. 10.1126/scirobotics.adf1080 PMC10169237 36791215

[B4] Chu J.-U. Moon I. Mun M.-S. (2006). “A supervised feature projection for real-time multifunction myoelectric hand control,” in 2006 International Conference of the IEEE Engineering in Medicine and Biology Society, New York, 3 September 2006 (IEEE), 2417–2420. 10.1109/IEMBS.2006.259659 17945714

[B5] Englsberger J. Ott C. Albu-Schäffer A. (2015). Three-dimensional bipedal walking control based on divergent component of motion. Ieee Trans. robotics 31, 355–368. 10.1109/tro.2015.2405592

[B6] Farkhatdinov I. Ebert J. Van Oort G. Vlutters M. Van Asseldonk E. Burdet E. (2019). Assisting human balance in standing with a robotic exoskeleton. IEEE Robotics automation Lett. 4, 414–421. 10.1109/lra.2018.2890671

[B7] Huo W. Moon H. Alouane M. A. Bonnet V. Huang J. Amirat Y. (2021). Impedance modulation control of a lower-limb exoskeleton to assist sit-to-stand movements. IEEE Trans. Robotics 38, 1230–1249. 10.1109/tro.2021.3104244

[B8] Kajita S. Morisawa M. Miura K. Nakaoka S. Harada K. Kaneko K. (2010). “Biped walking stabilization based on linear inverted pendulum tracking,” in 2010 IEEE/RSJ International Conference on Intelligent Robots and Systems, USA, 18-22 Oct. 2010 (IEEE), 4489–4496.

[B9] Karavas N. Ajoudani A. Tsagarakis N. Caldwell D. (2013).Human-inspired balancing assistance: application to a knee exoskeleton, 2013 IEEE international conference on robotics and biomimetics (ROBIO), 12-14 Dec. 2013, China. IEEE, 292–297.

[B10] Lippi V. Mergner T. (2020). A challenge: support of standing balance in assistive robotic devices. Appl. Sci. 10, 5240. 10.3390/app10155240

[B11] Mantoan A. Pizzolato C. Sartori M. Sawacha Z. Cobelli C. Reggiani M. (2015). Motonms: a matlab toolbox to process motion data for neuromusculoskeletal modeling and simulation. Source code Biol. Med. 10, 12–14. 10.1186/s13029-015-0044-4 26579208 PMC4647340

[B12] Millard M. Wight D. McPhee J. Kubica E. Wang D. (2009). Human foot placement and balance in the sagittal plane. 10.1115/1.4000193 20524724

[B13] Nishiwaki K. Kagami S. (2009). Online walking control system for humanoids with short cycle pattern generation. Int. J. Robotics Res. 28, 729–742. 10.1177/0278364908097883

[B14] Peng Z. Jiang S. Li J. (2017). “Control strategies for stability recovery of full lower limb exoskeleton robot based on plantar pressure,” in 2017 12th IEEE Conference on Industrial Electronics and Applications (ICIEA), USA, 18-20 June 2017 (IEEE), 1218–1223.

[B15] Postol N. Spratt N. J. Bivard A. Marquez J. (2021). Physiotherapy using a free-standing robotic exoskeleton for patients with spinal cord injury: a feasibility study. J. NeuroEngineering Rehabilitation 18, 180–210. 10.1186/s12984-021-00967-4 PMC8709973 34953501

[B16] Pratt J. Carff J. Drakunov S. Goswami A. (2006). “Capture point: a step toward humanoid push recovery,” in 2006 6th IEEE-RAS international conference on humanoid robots (IEEE), Italy, 04-06 December 2006 (IEEE), 200–207.

[B17] Pratt J. Chew C.-M. Torres A. Dilworth P. Pratt G. (2001). Virtual model control: an intuitive approach for bipedal locomotion. Int. J. Robotics Res. 20, 129–143. 10.1177/02783640122067309

[B18] Rajasekaran V. Aranda J. Casals A. Pons J. L. (2015). An adaptive control strategy for postural stability using a wearable robot. Robotics Aut. Syst. 73, 16–23. 10.1016/j.robot.2014.11.014

[B19] Rodríguez-Fernández A. Lobo-Prat J. Font-Llagunes J. M. (2021). Systematic review on wearable lower-limb exoskeletons for gait training in neuromuscular impairments. J. neuroengineering rehabilitation 18, 22–21. 10.1186/s12984-021-00815-5 PMC7852187 33526065

[B20] Shi D. Zhang W. Zhang W. Ding X. (2019). A review on lower limb rehabilitation exoskeleton robots. Chin. J. Mech. Eng. 32, 74–11. 10.1186/s10033-019-0389-8

[B21] Siviy C. Baker L. M. Quinlivan B. T. Porciuncula F. Swaminathan K. Awad L. N. (2023). Opportunities and challenges in the development of exoskeletons for locomotor assistance. Nat. Biomed. Eng. 7, 456–472. 10.1038/s41551-022-00984-1 36550303 PMC11536595

[B22] Stegall P. Winfree K. Zanotto D. Agrawal S. K. (2013). Rehabilitation exoskeleton design: exploring the effect of the anterior lunge degree of freedom. IEEE Trans. Robotics 29, 838–846. 10.1109/tro.2013.2256309

[B23] Stephens B. (2007). “Humanoid push recovery,” in 2007 7th IEEE-RAS International Conference on Humanoid Robots (IEEE), USA, December 1, 2007 (IEEE), 589–595.

[B24] Stephens B. J. Atkeson C. G. (2010). “Push recovery by stepping for humanoid robots with force controlled joints,” in 2010 10th IEEE-RAS International conference on humanoid robots (IEEE), USA, 6-8 Dec. 2010 (IEEE), 52–59.

[B25] Su D. Hu Z. Wu J. Shang P. Luo Z. (2023). Review of adaptive control for stroke lower limb exoskeleton rehabilitation robot based on motion intention recognition. Front. Neurorobotics 17, 1186175. 10.3389/fnbot.2023.1186175 PMC10350518 37465413

[B26] Sugiura S. Unde J. Zhu Y. Hasegawa Y. (2023a). Variable grounding flexible limb tracking center of gravity for sit-to-stand transfer assistance. IEEE Robotics Automation Lett. 9, 175–182. 10.1109/lra.2023.3328449

[B27] Sugiura S. Zhu Y. Huang J. Hasegawa Y. (2023b). Passive lower limb exoskeleton for kneeling and postural transition assistance with expanded support polygon. IEEE/ASME Trans. Mechatronics, 1–12. 10.1109/tmech.2023.3294255

[B28] Ugurlu B. Doppmann C. Hamaya M. Forni P. Teramae T. Noda T. (2015). Variable ankle stiffness improves balance control: experiments on a bipedal exoskeleton. IEEE/ASME Trans. mechatronics 21, 79–87. 10.1109/TMECH.2015.2448932

[B29] van der Kooij H. van Asseldonk E. H. Vlutters M. (2016). “Towards exoskeletons with balance capacities,” in Wearable Robotics: Challenges and Trends: Proceedings of the 2nd International Symposium on Wearable Robotics, WeRob2016, Segovia, Spain, October 18-21, 2016 (Springer), 175–179.

[B30] Wieber P.-B. (2006). “Trajectory free linear model predictive control for stable walking in the presence of strong perturbations,” in 2006 6th IEEE-RAS International Conference on Humanoid Robots (IEEE), Italy, 04-06 December 2006 (IEEE), 137–142.

[B31] Winter D. A. (1995). Human balance and posture control during standing and walking. Gait posture 3, 193–214. 10.1016/0966-6362(96)82849-9

[B32] Zhang L. Li Z. Hu Y. Smith C. Farewik E. M. G. Wang R. (2020). Ankle joint torque estimation using an emg-driven neuromusculoskeletal model and an artificial neural network model. IEEE Trans. Automation Sci. Eng. 18, 564–573. 10.1109/tase.2020.3033664

[B33] Zhou J. Yang S. Xue Q. (2021). Lower limb rehabilitation exoskeleton robot: a review. Adv. Mech. Eng. 13, 168781402110118. 10.1177/16878140211011862

